# Assessment of potential probiotic lactic acid bacteria in rice-based fermented products of Southern Assam, Northeast India

**DOI:** 10.3389/fmicb.2025.1536593

**Published:** 2025-06-18

**Authors:** Hanna Yumnam, Soumitra Nath, Pragya Chakraborty, Indu Sharma

**Affiliations:** ^1^Department of Microbiology, Assam University, Silchar, Assam, India; ^2^Department of Biotechnology, Gurucharan College, Silchar, Assam, India

**Keywords:** indigenous fermented products, probiotic bacteria, lactic acid, safety criteria, gut health

## Abstract

**Introduction:**

Indigenous ethnic fermented products have gained recognition as valuable sources of probiotic bacteria, which play a crucial role in maintaining the natural balance of the gut microbiota.

**Methods:**

Twenty-five samples of rice-based fermented products were analyzed, recovering 16 LAB isolates, preliminarily identified by morphological and biochemical tests, and further confirmed using 16S rRNA gene sequencing. Subsequent *in vitro* assessments, safety evaluations and technological properties were performed on all isolated strains.

**Results:**

The potential probiotic strains were able to tolerate low pH, gastric juice, bile salts, and pancreatin enzymes; however notable variations in resistance were also observed and several strains demonstrated a significant ability of the strains to adhere to intestinal epithelial cells. All the isolates lacked DNase and hemolytic activity, confirming the safety of the isolates. Antibiotic susceptibility profiles indicated no harmful resistance patterns and displayed significant antagonistic activity against test pathogens. Furthermore, the study reported the absence of technological properties, specifically in terms of proteolytic, lipolytic, and amylolytic activity.

**Discussion:**

Most of the isolated strains showed significantly significant results with p <0.05 and <0.01, reinforcing the probiotic potential and safety of the isolates. These promising isolated strains hold potential as safe probiotics, offering significant health benefits.

## 1 Introduction

Traditional knowledge from the ancient Indian culture is fundamentally based on fermented foods. This ground-breaking process of food fermentation has evolved and grown over the generations to conserve and fortify the existing food resources. India, the second largest rice grower in the world, has a long history of making traditionally fermented meals using rice that has a variety of flavors and textures related to cultural diversity, and is typically made by rural women using traditional village arts techniques (Ray et al., 2016). It is essential to undertake further in-depth studies and development to ensure the safety and nutritional value of these traditional fermented foods, especially as their production and consumption become increasingly common and associated with food security concerns. Fermented foods may also improve flavor, digestibility, nutritional value, and medicinal properties in addition to preservation (Jeyaram et al., 2009). Probiotics are live bacteria that, when present in adequate quantities, provide various health advantages to the host. As a result, they are frequently utilized to enhance the health of both humans and animals by modifying the gut microbiota (Saavedra et al., 2004). The most prevalent microorganisms usually found in rice-based fermented products contain lactic acid bacteria (LAB), including lactobacilli, bifidobacteria, yeasts, and molds, which are involved in fermentation (Satish Kumar et al., 2013).

Rice is the primary crop in Northeast India, and the consumption and production of rice-based fermented foods are prevalent among various communities in this region. The hill states are predominantly inhabited by tribal people with significant diversity. Assam, the second-largest state in Northeast India, is home to a wide range of ethnocultural groups. The geographical location and climatic conditions of this area facilitate the preparation of traditional products by these ethnic groups (Loying et al., 2024). This study addresses a significant research gap concerning the traditional fermented rice-based foods of Northeast India, particularly focusing on the diverse tribal communities in southern Assam. Despite the longstanding cultural and dietary importance of these foods, comprehensive studies evaluating their safety, nutritional value, and potential as probiotics are lacking. The study aims to isolate and characterize lactic acid bacteria strains from these traditional foods to assess their probiotic potential.

## 2 Materials and methods

### 2.1 Collection of the sample, bacterial isolation, and identification

#### 2.1.1 Criterion for sampling

A total of 25 samples were collected based on rice-based fermented products from five different ethnic groups of Southern Assam: Zeme Naga, Dimasa Kachari, Hmar, Karbi, and Tea Tribe, with 30 ml collected from each group. All the samples were collected before the distillation process. These traditional fermented products typically undergo natural fermentation at room temperature for up to 3–5 days, depending on the season. Fermentation usually takes 2–3 days in summer and 4–5 days during winter. This study did not include any fermented products sold at the local markets. The samples that were prepared traditionally were taken in sterilized vials and carried to the laboratory for microbiological sample investigation. The collected samples were stored in the fridge (about 4°C) before the analysis began.

#### 2.1.2 Isolation and biochemical characterization of LAB isolates

The samples were serially diluted (10^–3^–10^–5^) using a phosphate buffer saline (PBS) solution. Afterward, a 0.1 mL portion of each dilution was streaked onto de Man–Rogosa–Sharpe (MRS) agar medium (Himedia, India). Subsequently, the plates were incubated at 37°C for 24–48 h (Sreenadh et al., 2022). Following the incubation period, individual and distinct colonies were sub-cultured and preliminarily identified based on their colony morphology, cultural characteristics, and Gram staining. Biochemical tests were conducted for all isolated bacteria, which included the Indole test, Methyl red test, Voges–Proskauer test, Catalase test, Citrate utilization test, and Oxidase test (Cappuccino and Sherman, 2005).

#### 2.1.3 Molecular characterization of the isolates

Genomic DNA was extracted from the bacterial culture using a QIAGEN DNeasy Ultraclean Microbial kit (Cat. No/ID: 12224- 50) (QIAGEN, Germany, USA) as per the manufacturer’s instructions. Its quality was evaluated on 1.0% agarose gel, and a single band of high-molecular-weight DNA was observed. The fragment of the 16S rRNA gene was amplified by primers 16SF-27F (5′-AGAGTTTGATCCTGGCTCAG-3′) and 16SR-1492R (5′-CGGTTACCTTGTTACGACTT-3′) (Suardana, 2014). The conditions used for amplification were set as initial denaturation for 3 min at 95°C, 35 cycles of denaturation for 30 s at 95°C, annealing for 30 s at 50°C, and elongation for 45 s at 72°C with a final extension for 3 min at 72°C. A single discrete PCR amplicon band of 1,500 bp was observed when resolved on agarose gel. The PCR amplicon was purified to remove contaminants using the QIAGEN QIAquick PCR Purification Kit (Cat. No/ID: 28104) (QIAGEN, Germany, USA). The sequencing of the 16S rRNA gene was carried out at Centyle Biotech, New Delhi, India, using a BDT v3.1 cycle sequencing kit on an ABI 3720xl Genetic Analyzer. A consensus sequence was generated from forward and reverse sequence data using aligner software (Nath et al., 2018). The 16S rRNA gene sequence was compared with reference bacteria from the National Centre for Biotechnological Information (NCBI) GenBank, using the nucleotide BLAST algorithm available at http://www.ncbi.nlm.nih.gov/blast for highly significant matches. All the sequences were aligned using Clustal-W, and a phylogenetic tree was constructed using RAxML. The tree was generated with a GAMMA-based likelihood approach and the Rapid Hill-Climbing algorithm, with a focus on optimizing branch lengths jointly (Izquierdo-Carrasco et al., 2011). All analysis was conducted using the Geneious R8 software package, developed by Biomatters Ltd., Auckland, New Zealand.

### 2.2 Probiotic assessment of isolated bacterial strains

#### 2.2.1 Acid tolerance test

The pH tolerance of the isolated strain was determined by inoculating the isolates in MRS broth at pH 2.0 and 3.0, separately (Khushboo et al., 2023; Chen et al., 2022). Bacterial strains were also inoculated at pH 7.0 in MRS broth, which acted as a control. The pH of the broth was adjusted using 1 N HCl. All the tubes were incubated at 37°C for 3 h, and the optical density (OD) was measured at 600 nm.

#### 2.2.2 Simulated gastric juice tolerance test

This assay evaluates the ability of isolated strains to withstand harsh gastric conditions. The test was conducted according to the protocol outlined by Urcan et al. (2024), with slight modifications. Gastric juice was prepared by dissolving 3 g/L pepsin (Himedia, India), 7 mM KCL, 45 mM NaHCO3, and 125 mM NaCl, adjusting to a pH of 3. Overnight bacterial broth cultures were centrifuged at 10,000 rpm for 15 min to pellet the bacteria. The bacterial pellets were then re-suspended in 10 mL of gastric juice and 10 mL of PBS buffer (pH 7.2) without gastric juice (pH 7.0) acted as a control. The growth of each strain was monitored by measuring the OD at 600 nm after 3 h of incubation. This allowed for the assessment of bacterial viability and growth in the presence of simulated gastric juice.

#### 2.2.3 Bile tolerance test

The bile tolerance test was conducted according to the method described by Nath et al. (2020a). One hundred microliters of overnight-grown bacterial suspension was inoculated into MRS broth containing 0.3 and 0.5% bile salt, respectively. Samples were also inoculated in MRS broth without bile, which acted as a control. All the test tubes were incubated at 37°C, and the OD was recorded at a wavelength of 600 nm after 4 h of incubation.

#### 2.2.4 Pancreatin tolerance test

Overnight bacterial cultures were introduced into two types of MRS broth: one containing 0.5% (v/w) pancreatin (Himedia, India) and the other without pancreatin, which served as the control. All the inoculated broths were placed in a shaker incubator for 2 min, during which initial absorbance readings were recorded. Subsequently, they were further incubated for 24 h at 37°C, after which final absorbance measurements were taken at 600 nm (Nath et al., 2020a).

#### 2.2.5 Cell surface hydrophobicity assay

The assessment of cell-surface hydrophobicity was conducted using methods described by Rosenberg et al. (1980). This evaluation typically involves techniques such as contact angle measurement and Microbial Adhesion to Hydrocarbons (MATH) (Rosenberg et al., 1980; Sreenadh et al., 2022). n-Hexadecane and xylene were selected for their distinct hydrophobic properties to assess cell surface hydrophobicity and its impact on intestinal adhesion. To perform the test, an overnight-grown bacterial culture in MRS broth was centrifuged at 12,000 rpm for 5 min to recover the bacterial cells. The pellets were then washed twice with PBS buffer (pH 7.2) and suspended in 6 mL of PBS buffer. The initial absorbance (OD_initial) was measured at 600 nm. Subsequently, 2 mL of the bacterial suspension was mixed with 0.5 mL of hydrocarbons (n-hexadecane and xylene) and vortexed for 2 min. The mixture was incubated for 1 h at 37°C, after which the aqueous phase was carefully removed, and the final absorbance (OD_final) was measured at 600 nm (Nath et al., 2020a). The rate of cell surface hydrophobicity was calculated using the formula:


$$\mathrm{Rate}\mathrm{of}\mathrm{cell}\mathrm{surface}\mathrm{hydrophobicity}\left(\%\right)=\frac{O\,D_{i\,n\,i\,t\,i\,a\,l}-O\,D_{f\,i\,n\,a\,l}}{O\,D_{i\,n\,i\,t\,i\,a\,l}}\mathrm{X100}$$

#### 2.2.6 Auto-aggregation assay

The specific cell-cell interaction or auto-aggregation was assessed using the method outlined by Xu et al. (2009). Initially, cell pellets were obtained from an overnight culture grown in MRS broth by centrifugation at 2,000 × g force for 10 min at 4°C. The pellets were washed twice with PBS solution and resuspended in 6 mL of the same solution. An initial absorbance (OD_*initial*_) was taken at 600 nm. Subsequently, the cells were subjected to incubation at 40°C for 2 h, following which the final absorbance (OD_*final*_) was measured to determine the percentage of cellular auto-aggregation.


$$\mathrm{Rate}\mathrm{of}\mathrm{Auto}-\mathrm{aggregation}\left(\%\right)=\frac{O\,D_{i\,n\,i\,t\,i\,a\,l}-O\,D_{f\,i\,n\,a\,l}}{O\,D_{i\,n\,i\,t\,i\,a\,l}}\mathrm{X100}$$

### 2.3 *In vitro* biofilm assay

#### 2.3.1 Congo red agar method

Biofilm formation by the isolated 16 strains was determined by the Congo red agar (CRA) (Himedia, India) method, following the protocol as described by Hazarika et al. (2021). Briefly, Brain Heart Infusion agar (BHI) (Himedia, India) (37 g/L) and sucrose (2 g/L) were mixed to prepare Congo red agar medium. The mixture was then autoclaved, and Congo red stain (0.8 g/L) was added to it. Afterward, the medium was poured onto Petri plates and allowed to solidify. The isolated organisms (∼1 × 10^8^ CFU/mL) were streaked onto the plates and incubated aerobically at 37°C for 24 h. The appearance of black, dry, crystalline colonies on the CRA plates was classified as a strong biofilm producer, while red or pink colonies were classified as weak biofilm producers. Dark colonies without crystalline morphology were classified as intermediate biofilm producers.

#### 2.3.2 Tissue culture plate method

The biofilm formation was also assessed using the tissue culture plate (TCP) method (Christensen et al., 1985; Hassan et al., 2011). Briefly, test organisms (∼1 × 10^8^ CFU/mL) were inoculated into TSB with 1% glucose and incubated for 24 h at 37°C. After the incubation period, 200 μL of each TSB glucose containing the isolated strains was placed into separate wells of sterilized 96-well polystyrene plates with a flat bottom for tissue cultivation. The plates underwent incubation and were maintained at 37°C for 24 h. Following incubation, each of the wells’ contents was gently tapped out and rinsed 2–3 times with 0.2 mL PBS (pH 7.2) to remove the free-floating bacteria. The well-developed bacterial biofilm was maintained with 2% sodium acetate and dyed with 0.1% crystal violet. After removing the excessive stain with distilled water, the plates were allowed to air dry naturally. The experiment was carried out in duplicate. The final reading was taken by using an ELISA reader at 600 nm wavelength, and the stained adherent biofilm’s optical density (OD) was determined.

### 2.4 Safety assessment

#### 2.4.1 Antibiotic susceptibility profile

All identified isolates from ethnic rice-based fermented products were subjected to antibiogram susceptibility profiling using the Kirby-Bauer disc diffusion method on Mueller-Hinton agar (MHA) media (Himedia, India) (Bauer et al., 1966; Nath et al., 2021). Bacterial suspensions were prepared by mixing colonies (1.5 × 10^8^ CFU/mL) with saline and adjusting the turbidity to 0.5 McFarland standard. This standardized suspension was then used to prepare lawn cultures on MHA plates. The antibiotics used in the present study were procured from Himedia, India which includes erythromycin (15 mcg), ciprofloxacin (5 mcg), gentamicin (10 mcg), chloramphenicol (30 mcg), amikacin (30 mcg), vancomycin (10 mcg), oxacillin (1 mcg), co-trimoxazole (25 mcg), meropenem (10 mcg), and norfloxacin (10 mcg). Antibiotic discs were placed on freshly prepared lawns of each isolate on MHA plates and incubated at 37°C for 24–48 h. The diameter of the inhibition zones was measured and classified according to the guidelines of the Clinical and Laboratory Standards Institute (CLSI, 2025). The reference strain used for quality assurance was *E. coli* ATCC 25922. The statistical program SPSS version (IBM SPSS Statistics 29.0.0.0) was used to analyze the antibiotic susceptibility exhibited by isolated lactic acid bacteria. A chi-square test was performed, where *p* < 0.05 was observed as statistically significant.

#### 2.4.2 DNase activity

The test isolates (∼1 × 10^8^ CFU/mL) were streaked onto DNase agar plates and subsequently incubated aerobically at temperatures ranging from 37 to 40°C for a period of 72 h. Following incubation, the plates were treated by flooding them with a 3% HCl solution for 5–8 min. The plates were then observed for the presence of clear zones surrounding the colonies as an indication of DNase activity (Rastogi et al., 2019; Sreenadh et al., 2022).

#### 2.4.3 Haemolytic activity

Hemolytic activity was assessed using blood agar plates supplemented with 5% sheep blood on agar. The test isolate (∼1 × 10^8^ CFU/mL) was streaked onto the surface of freshly prepared blood agar plates and subsequently incubated at temperatures ranging from 37 to 40°C for 24–48 h. The plates were then observed for the presence of hemolysis, categorized as alpha (α)—partial hemolysis, beta (β)—complete hemolysis, or gamma (γ)—no hemolysis (Nath et al., 2020b).

#### 2.4.4 Antagonistic activity of isolates

Antagonism activity of all the isolated bacteria was done using the well diffusion method (Ridwan et al., 2008; Sreenadh et al., 2022). Test microorganisms were obtained from the culture collection of the Institutional Biotech Hub, Gurucharan College, Silchar, which includes *Bacillus cereus* strain GCC_21R1, *Bacillus nealsonii* strain GCC_21R8, *Enterobacter bugandensis* strain GCC_21R10, *E. coli* ATCC 25922, and *Staphylococcus aureus* ATCC 23235. At first, 100 μL (1.5 × 10^8^ CFU/mL) of the test pathogen was spread onto the surface of MHA media (Himedia, India) using a sterile spreader. Wells were made on MHA plates using a cork borer, and the wells were filled with 50 μL (∼7.5 × 10^7^ CFU/mL) of overnight-grown isolates. The plates were incubated at 37°C for 24 h, and after the incubation period, the diameter of the zone of inhibition was measured.

### 2.5 Evaluation of technological properties

#### 2.5.1 Proteolytic activity

Proteolytic activity was assessed using an agar medium consisting of skimmed milk powder (10%) and agar (2%). Wells were carefully formed in the agar, into which twenty microliters of bacterial cultures (3 × 10^7^ CFU/mL) were introduced. The plates were then subjected to incubation at 37°C for 48–72 h. The presence of a clear zone surrounding the wells served as an indicator of positive proteolytic activity (Raveschot et al., 2020; Sreenadh et al., 2022).

#### 2.5.2 Lipolytic activity

The isolates (∼1 × 10^8^ CFU/mL) were streaked onto tributyrin agar plates and incubated at 37°C for 72–96 h. The presence of a clear zone surrounding the colonies was interpreted as indicative of positive lipolytic activity (Aspri et al., 2017).

#### 2.5.3 Amylolytic activity

The amylolytic activity was assessed by spot-inoculating the test isolate (∼1 × 10^8^ CFU/mL) onto the surface of Luria-Bertani (LB) agar (Himedia, India) supplemented with 20 g/L of soluble starch, followed by incubation at 37°C for 72 h. Subsequently, iodine solution (1%) was flooded onto the inoculated plates. The presence of a halo zone surrounding the colonies upon observation confirmed positive results indicative of amylolytic activity (do Espirito-Santo et al., 2014; Sreenadh et al., 2022).

### 2.6 Statistical analysis

All experiments were performed in triplicate, and the statistical analysis was carried out using Microsoft Excel 2007 and SPSS version 16. The OD values are expressed as the mean ± standard deviation (SD) of absorbance units. Statistical comparisons were made using the paired *t*-test, and differences were considered statistically significant at *p* < 0.01 and *p* < 0.05.

## 3 Results

### 3.1 Identification of bacteria

#### 3.1.1 Morphological and biochemical identification

In the present study, a total of 16 lactic acid bacterial strains were isolated from rice-based fermented products of ethnic groups in Southern Assam, India. Morphological characterization revealed that all isolates were Gram-positive, displaying bacilli morphology and some exhibiting cocci shape. Biochemical tests were conducted to characterize the isolates (Table 1) further. Results indicated that the isolates tested negative for the indole, Voges-Proskauer, citrate utilization, catalase, and oxidase tests. However, variability was observed in the methyl red test results among the isolates.

**TABLE 1:** Morphological and biochemical characteristics of the isolated bacteria“+” indicates positive result and “-” indicates a negative result

Isolates	Morphological characteristics		Gram staining		Biochemical characteristics						Identified species	GenBank accession no.
	Colony color	Surface	Color	Shape	Indole test	Methyl red test	Voges -proskauer test	Citrate utilization test	Catalase test	Oxidase test		
DMK01	Off-white	Creamy	Purple	Spherical	–	+	–	–	–	–	*Pediococcus pentosaceus*	OR607371
DMK02	Off-white	Creamy	Purple	Spherical	–	+	–	–	–	–	*Pediococcus pentosaceus*	OR607372
DMK03	Off-white	Creamy	Purple	Rod-shaped	–	–	–	–	–	–	*Bacillus sp. (in firmicutes)*	OR594337
TT02	Off-white	Creamy	Purple	Rod-shaped	–	–	–	–	–	–	*Limosilactobacillus fermentum*	OR607381
TT03	Off-white	Granular	Purple	Rod-shaped	–	–	–	–	–	–	*Lactiplantibacillus plantarum*	OR607382
TT04	Off-white	Granular	Purple	Rod-shaped	–	–	–	–	–	–	*Lactiplantibacillus plantarum*	OR607383
K101	Off-white	Creamy	Purple	Rod-shaped	–	–	–	–	–	–	*Limosilactobacillus fermentum*	OR607377
K102	Off-white	Creamy	Purple	Spherical	–	+	–	–	–	–	*Pediococcus pentosaceus*	OR607378
K104	Off-white	Granular	Purple	Rod-shaped	–	–	–	–	–	–	*Lactobacillus plantarum*	OR607379
K105	Off-white	Creamy	Purple	Rod-shaped	–	+	–	–	–	–	*Priesta endophytica*	OR607380
HM01	Off-white	Creamy	Purple	Rod-shaped	–	+	–	–	–	–	*Priesta endophytica*	OR591461
HM03	Off-white	Creamy	Purple	Rod-shaped	–	–	–	–	–	–	*Lactiplantibacillus pentosus*	OR607375
HM05	Off-white	Creamy	Purple	Rod-shaped	–	+	–	–	–	–	*Limosilactobacillus fermentum*	OR607376
ZN01	Off-white	Granular	Purple	Rod-shaped	–	–	–	–	–	–	*Lactiplantibacillus plantarum*	OR607373
ZN04	Off-white	Creamy	Purple	Rod-shaped	–	–	–	–	–	–	*Lactiplantibacillus pentosus*	OR592093
ZN06	Off-white	Granular	Purple	Rod-shaped	–	+	–	–	–	–	*Lactiplantibacillus plantarum*	OR607374

#### 3.1.2 Molecular characterization and phylogenetic analysis

**FIGURE 1:**
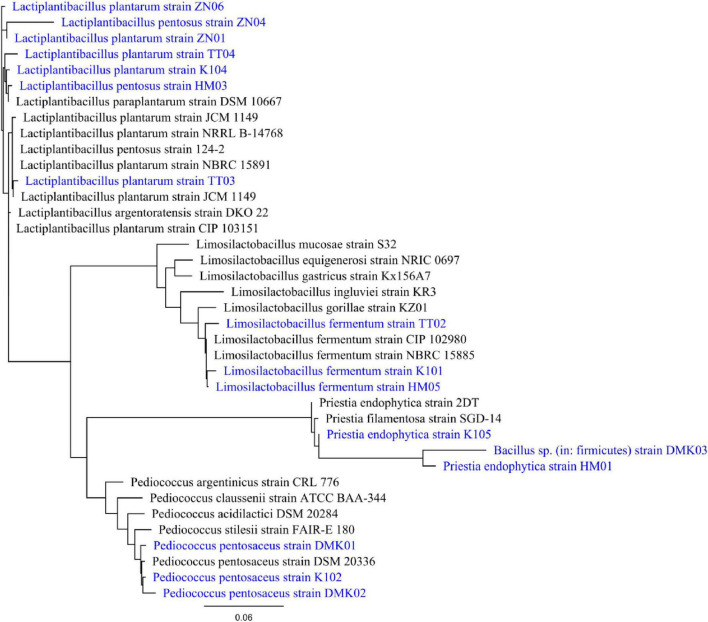
Phylogenetic tree of isolates DMK01, DMK02, DMK03, TT02, TT03, TT04, K101, K102, K104, K105, HM01, HM03, HM05, ZN01, ZN04 and ZN06. It is generated by aligning 16S rRNA gene sequence of the isolates with the database sequence, and the phylogenetic tree was constructed using RAxML. The tree was generated with a GAMMA-based likelihood approach and the Rapid Hill-Climbing algorithm.

The BLAST results indicated that the 16S rRNA gene sequence of the isolated bacteria shares 98–99% identity and covers 98% of the query with isolates stored in GenBank. The phylogenetic tree, depicted in Figure 1, was constructed using RaXML, illustrating the degree of relatedness with the sequences retrieved from the database. This tree was generated employing a likelihood approach based on the GAMMA model and the efficient Rapid Hill-Climbing algorithm. The GAMMA model provided highly accurate parameter estimates, with precision up to 0.1000000000 Log Likelihood units, while the Rapid Hill-Climbing mode optimized branch length. All 16S rRNA gene sequences have been submitted to NCBI GenBank, and the corresponding accession numbers are listed in Table 1.

### 3.2 Probiotic assessment of isolated bacterial strains

#### 3.2.1 Acid tolerance test

**FIGURE 2:**
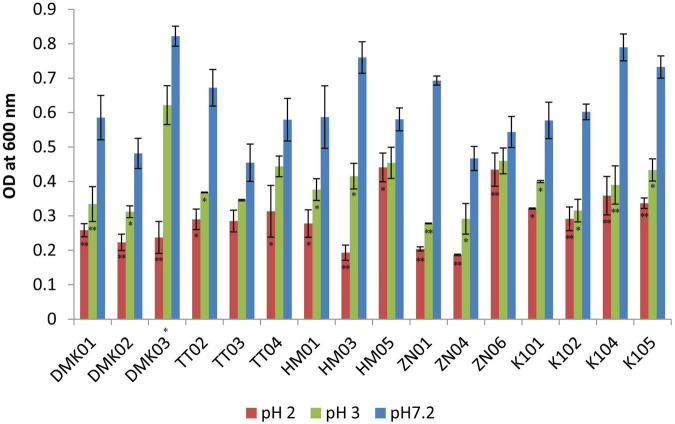
Acid tolerance test for isolated strains at pH 2.0 and pH 3.0, compared with control isolates at pH 7.2. OD values are presented as mean ± standard deviation (SD) of absorbance units. Statistical analysis was performed using the paired *t*-test. “*” and “**” indicate significance levels of *p* < 0.05 and *p* < 0.01, respectively.

The acid tolerance test demonstrated significant differences in the survival and growth capacity of the isolated LAB strains under acidic conditions (pH 2.0 and 3.0) relative to the control at pH 7.2, as determined by optical density at 600 nm (OD_600_) (Figure 2). While strains such as TT03 and TT04 showed statistically significant reductions in growth at low pH (*P* < 0.05), their absolute OD values under acidic stress were comparatively lower than those of other isolates. In contrast, strains HM05, ZN06, and K101 exhibited both statistically significant survival and quantitatively higher growth under acidic conditions, marking them as the most acid-tolerant candidates. Specifically, HM05 recorded OD values of 0.580 ± 0.032 at pH 7.2, 0.454 ± 0.033 at pH 3.0, and 0.441 ± 0.041 at pH 2.0, with a minimal reduction of 0.139 at the most acidic condition. ZN06 maintained high viability with OD values of 0.544 ± 0.047, 0.460 ± 0.038, and 0.434 ± 0.048 at pH 7.2, 3.0, and 2.0, respectively, corresponding to a small decrease of 0.110. K101 showed moderate acid tolerance with OD readings of 0.577 ± 0.042 at pH 7.2, 0.400 ± 0.003 at pH 3.0, and 0.321 ± 0.002 at pH 2.0, indicating a larger but still acceptable decline of 0.256. These results suggest that while TT03 and TT04 are statistically responsive to acid stress, HM05, ZN06, and K101 are superior in actual growth retention, supporting their probiotic potential for surviving harsh gastric conditions.

#### 3.2.2 Simulated gastric juice tolerance test

**FIGURE 3:**
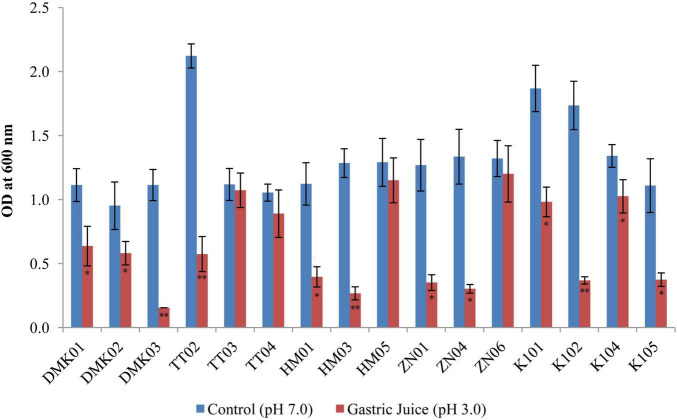
Bar graph showing simulated gastric tolerance of the isolates at pH 3.0 after 3 hours of incubation, compared with control isolates without gastric juice (at pH 7.0). OD values are presented as mean ± standard deviation (SD) of absorbance units. Statistical analysis was performed using the paired *t*-test. “*” and “**” indicate significance levels of *p* < 0.05 and *p* < 0.01, respectively.

The simulated gastric juice tolerance test revealed significant variability in the survival rates of LAB isolates after 3 h of incubation at pH 3.0 compared to neutral control conditions at pH 7.0 (Figure 3). Among the tested strains, HM05 and ZN06 exhibited the highest resistance to gastric acidity. HM05 showed a mean OD_600_ of 1.291 ± 0.183 under control conditions and 1.150 ± 0.176 in simulated gastric juice, with a minimal reduction of 0.141, which was statistically non-significant (*P* > 0.05). ZN06 demonstrated similar resilience, with OD values of 1.321 ± 0.191 and 1.201 ± 0.220 under control and acidic conditions, respectively, reflecting a small decrease of 0.120 (*P* > 0.05). Although TT03 exhibited a high OD under both conditions, 1.118 ± 0.125 in control and 1.073 ± 0.135 in gastric juice, the difference was statistically significant (*P* > 0.05), suggesting moderate acid-induced stress. In contrast, isolates such as DMK03, HM03, and K104 showed significantly lower survival in acidic conditions, with DMK03 maintaining a constant but low OD of 0.155 ± 0.000, and HM03 showing a dramatic decline from 1.285 ± 0.111 to 0.268 ± 0.052 (*P* < 0.01). These results highlight HM05 and ZN06 as the most robust strains against simulated gastric juice, underscoring their potential viability during gastrointestinal passage.

#### 3.2.3 Bile tolerance test

**FIGURE 4:**
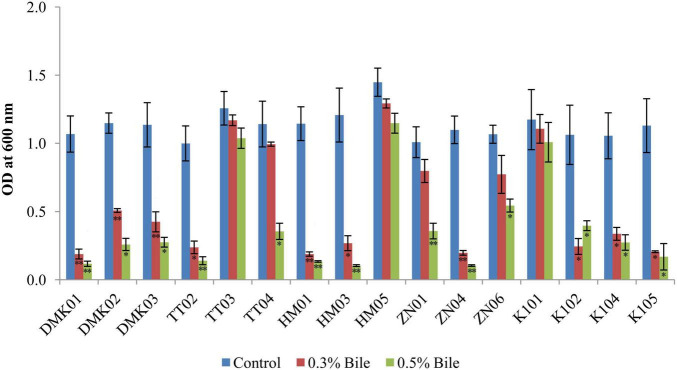
Tolerance of isolated strains in the presence of 0.3 and 0.5% bile salt, compared with a control set without bile amendment. OD values are expressed as mean ± standard deviation (SD) of absorbance units. Statistical analysis was performed using the paired *t*-test. “*” and “**” indicate significance levels of *p* < 0.05 and *p* < 0.01, respectively.

The bile tolerance assay revealed considerable variability in the survival of LAB isolates under increasing bile salt concentrations (0.3 and 0.5%) compared to control conditions (without bile). Under control conditions, HM05 showed the highest mean OD_600_ (1.448 ± 0.103), followed by TT03 (1.396 ± 0.064) and TT04 (1.321 ± 0.058) (Figure 4). In 0.3% bile, HM05 retained substantial viability with a mean OD of 1.292 ± 0.033, representing a mean reduction of 0.156, which was not statistically significant (*P* > 0.05). TT03 also maintained notable growth (OD: 1.169 ± 0.039), showing a mean difference of 0.227 compared to the control (P < 0.05), indicating statistically significant but relatively low reduction. Similarly, K101 exhibited good bile tolerance with an OD of 1.173 ± 0.107 at 0.3% bile (reduction of 0.168 from control; *P* < 0.05), while ZN06 showed moderate tolerance (OD: 0.772 ± 0.132, reduction of 0.417; P < 0.01). At 0.5% bile, further reductions were noted across most isolates. However, K101 demonstrated strong resilience with an OD of 1.008 ± 0.144, corresponding to a total reduction of 0.265 from the control (*P* < 0.05), and only 0.165 from the 0.3% level. HM05 also maintained a high OD of 1.147 ± 0.069 at 0.5% bile, with a total mean difference of 0.301 from the control (*P* < 0.05), indicating robust bile resistance. TT03 retained viability (OD: 0.994 ± 0.015), with a cumulative reduction of 0.402 from control (*P* < 0.01), reflecting good adaptation despite significant decline. Conversely, isolates DMK01 and ZN04 exhibited a drastic reduction in OD (*P* < 0.01), confirming poor tolerance. These findings highlight HM05, K101, and TT03 as the most bile-tolerant strains, effectively maintaining growth across 0.3 and 0.5% bile concentrations, thereby affirming their probiotic viability.

#### 3.2.4 Pancreatin tolerance test

**FIGURE 5:**
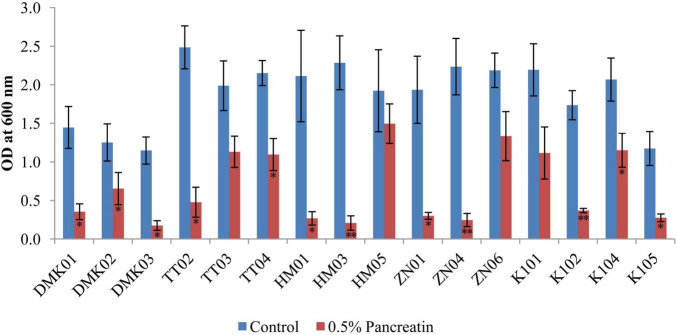
Bar graph illustrating the tolerance of isolated strains in the presence of 0.5% pancreatin, compared with the control set without pancreatin. OD values are presented as mean ± standard deviation (SD) of absorbance units. Statistical analysis was performed using the paired *t*-test. “*” and “**” indicate significance levels of *p* < 0.05 and *p* < 0.01, respectively.

The pancreatin tolerance test demonstrated significant variability in the ability of bacterial isolates to survive in the presence of 0.5% pancreatin (Figure 5). Among all tested strains, HM05 exhibited the highest pancreatin resistance, maintaining a mean OD of 1.496 ± 0.256 under treatment conditions versus a control mean of 1.922 ± 0.421, with a mean reduction of 0.426 (*P* < 0.05), indicating substantial tolerance. Similarly, ZN06 also retained strong viability with a pancreatin OD of 1.334 ± 0.319, compared to 2.188 ± 0.171 in the control, resulting in a moderate reduction of 0.854 (*P* < 0.05). Strains such as TT03 (pancreatin OD: 1.131 ± 0.201 vs. control: 1.987 ± 0.327, OD = 0.856, *P* < 0.05), K104 (1.151 ± 0.202 vs. 2.067 ± 0.278, OD = 0.916, *P* < 0.05), and K101 (1.115 ± 0.313 vs. 2.193 ± 0.325, OD = 1.078, *P* < 0.05) also showed acceptable tolerance with statistically significant but relatively smaller reductions. In contrast, isolates DMK03 and HM03 showed the poorest survival. DMK03 declined sharply from a control mean OD of 1.147 ± 0.179 to 0.175 ± 0.061 under pancreatin, yielding a reduction of 0.972 (*P* < 0.01). HM03 experienced a similar steep decline from 2.285 ± 0.345 to 0.207 ± 0.093, corresponding to a dramatic drop of 2.078 (*P* < 0.01), reflecting pronounced pancreatin sensitivity. These findings suggest that HM05 and ZN06 are the most pancreatin-tolerant isolates, with the least reduction in growth under enzymatic stress, while DMK03 and HM03 are the most susceptible, thereby helping to distinguish strains with enhanced resilience for potential probiotic applications.

#### 3.2.5 Cell surface hydrophobicity assay

The isolated bacteria showed a range of hydrophobicity levels towards hydrocarbons, demonstrating low to medium to higher affinities, meaning the isolated bacterial strains displayed a broad spectrum of hydrophobic interactions with hydrocarbons, indicating differences in their cell surface properties (Table 2). Isolate K104, TT03, K101, HM05, TT04, and TT02 exhibited high hydrophobicity percentages toward the tested hydrocarbons, with values ranging from 51.3 to 52.9% for n-hexadecane and 41.1–58.9% for xylene. However, samples such as ZN04, HM03, DMK01, DMK02, ZN01, ZN06, K105, HM01, DMK03, and TT04 demonstrated relatively lower levels of hydrophobicity, with percentages ranging from 10.1 to 20.6% for both hydrocarbons.

**TABLE 2:** Results of cell surface hydrophobicity and cellular auto-aggregation of isolated bacteria.

Isolates	Cell surface hydrophobicity (%)		Cellular auto-aggregation (%)
	n-hexadecane	Xylene	
DMK01	19.2%	15.1%	18.5%
DMK02	19.2%	15.1%	18.5%
DMK03	14.2%	14.6%	11.6%
HM01	11.3%	10.1%	13.1%
HM03	11.3%	14.6%	11.2%
HM05	52.9%	46.3%	41.2%
ZN01	11.3%	10.1%	13.1%
ZN04	11.3%	14.6%	11.2%
ZN06	11.3%	10.1%	13.1%
K101	52.9%	46.3%	41.2%
K102	19.2%	15.1%	18.5%
K104	51.3%	58.9%	23.8%
K105	11.3%	10.1%	13.1%
TT02	52.9%	46.3%	41.2%
TT03	51.3%	58.9%	23.8%
TT04	51.3%	58.9%	23.8%

#### 3.2.6 Auto-aggregation assay

Isolates HM05, TT02, and K101 exhibited the highest levels of cellular auto-aggregation, with percentages reaching 41.2% of samples. This indicates a significant propensity for these bacterial strains to aggregate. Similarly, isolates TT04, K104, and TT03 also demonstrated relatively high auto-aggregation rates, at 23.8%. However, the isolates DMK03, HM03, ZN04, ZN06, ZN01, HM01, DMK01, DMK02, K102, and K105 displayed comparatively lower levels of cellular auto-aggregation, ranging from 11.2 to 18.5% (Table 2). These findings highlight the diversity in cellular auto-aggregation capabilities among the isolated bacterial strains, which could have implications for various microbial processes and interactions within the intestinal epithelium.

### 3.3 *In vitro* biofilm assay

**FIGURE 6:**
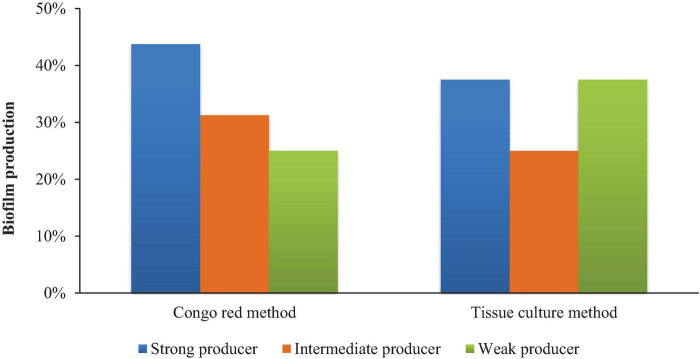
Comparison of biofilm formation using the CRA (Crystal Violet Assay), and TCP (Tissue Culture Plate) methods

The biofilm activity of the isolates was examined *in vitro* using the CRA method and the TCP technique. The CRA method revealed that 43.75% (7/16) of isolates were strong biofilm producers, 31.25% (5/16) were intermediate, and 25% were non-biofilm producers. While the TCP method revealed that 37.5% (6/16) were strong biofilm producers, 25% were moderate biofilm producers, and 37.5% showed no biofilm production (Figure 6).

### 3.4 Safety assessment

#### 3.4.1 Antibiotic susceptibility profile

The antimicrobial susceptibility profiling of 16 bacterial isolates against 10 antibiotics showed varied resistance and susceptibility patterns. In the present study, Erythromycin exhibited intermediate susceptibility in 62.5% of isolates, while 12.5% were resistant. Co-trimoxazole and Oxacillin showed 100% resistance. Gentamicin was effective in 93.75% of isolates, with only 6.25% showing intermediate susceptibility. Chloramphenicol exhibited varied results with 68.75% sensitivity, 18.75% intermediate, and 12.5% resistance. Amikacin showed 87.5% sensitivity, while 12.5% were resistant. Norfloxacin revealed 81.25% sensitivity, while 18.75% were resistant. Vancomycin exhibited 68.75% sensitivity, 18.75% intermediate, and 12.5% resistance. Meropenem was effective in 75% of isolates, with 18.75% intermediate and 6.25% resistant. Ciprofloxacin demonstrated sensitivity in 50%, intermediate in 31.25%, and resistance in 18.75%. The control strain (*E. coli* ATCC 25922) showed susceptibility patterns, with resistance for Erythromycin (18 mm) and Ciprofloxacin (20 mm). The obtained results were found to be statistically significant at *p* < 0.05 and are represented in Table 3.

**TABLE 3:** Antibiotic susceptibility profiles of bacterial isolates.

Bacterial isolates	Zone of inhibition (in mm)									
	Erythromycin	Co-trimoxazole	Gentamicin	Chloram phenicol	Amikacin	Norfloxacin	Vancomycin	Oxacillin	Meropenem	Cipro floxacin
DMK01	19 (I)	0	19(S)	22 (S)	20(S)	18(S)	19(S)	0	21(R)	12(R)
DMK02	19(I)	0	19(S)	17(I)	21(S)	20(S)	20(S)	0	24(S)	14(R)
DMK03	23(S)	0	20(S)	0	0	0	0	0	18(R)	0
HM01	14(I)	0	21(S)	0	0	0	19(S)	0	19(R)	26(S)
HM03	18(I)	0	20(S)	21(S)	19(S)	19(S)	15(I)	0	22(I)	28(S)
HM05	24(S)	0	20(S)	21(S)	15(I)	20(S)	15(I)	0	24(S)	28(S)
K101	23(S)	0	14(I)	17(I)	20(S)	17(S)	18(S)	0	21(I)	16(R)
K102	24(S)	0	20(S)	21(S)	19(S)	24(S)	12(R)	0	22(I)	24(I)
K104	15(I)	0	20(S)	21(S)	19(S)	18(S)	12(R)	0	23(S)	20(R)
K105	0	0	0	22(S)	21(S)	12(R)	0	0	18(R)	28(S)
TT02	0	0	19(S)	0	0	0	20(S)	0	20(I)	22(I)
TT03	23(S)	0	19(S)	12 (R)	14(R)	22(S)	19(S)	0	23(S)	15(R)
TT04	21(I)	0	21(S)	22(S)	16(I)	22(S)	19(S)	0	24(S)	22(I)
ZN01	20(I)	0	20(S)	15(I)	15(I)	20(S)	21(S)	0	20(I)	24(I)
ZN04	20(I)	0	20(S)	15(I)	14(R)	18(S)	20(S)	0	20(I)	23(I)
ZN06	24(S)	0	21(S)	22(S)	19(S)	18(S)	14(R)	0	29(S)	22(I)
*E. coli* ATCC25922	18 (R)	23 (S)	19 (S)	21(S)	19(S)	24(R)	17(R)	0	28(S)	20(R)

S, susceptible; I, intermediate; R, resistant (classified according to CLSI guidelines based on zone diameter breakpoints (CLSI 2025).

#### 3.4.2 DNase activity

All the tested isolates showed negative results for DNase activity. This is evidenced by the absence of any observable zone around the colonies of the test isolates upon flooding the DNase agar plates with 3% HCl.

#### 3.4.3 Haemolytic activity

The absence of a discernible zone of hemolysis surrounding the colonies of the tested strains on the blood agar plates suggests that the isolated strains do not exhibit any hemolytic activity. This result indicates a lack of red blood cell (RBC) breakdown, consistent with gamma hemolysis.

#### 3.4.4 Antagonistic activity

The antagonistic activity of the bacterial isolates was assessed using the agar well diffusion method against six test bacterial strains, and the results are summarized in Table 4. The inhibition zones varied widely across the isolates and target bacteria, indicating differing antimicrobial efficacies. Among the isolates, HM05, K101, and TT02 exhibited the most consistent and broad-spectrum antimicrobial activity. HM05 displayed strong inhibition against *E. bugandensis* (20.33 ± 1.53 mm) and *B. cereus* (20.67 ± 0.58 mm), along with substantial activity against *E. coli*, *S. aureus*, and *B. nealsonii*. Similarly, K101 showed significant inhibition against *E. bugandensis* (21.67 ± 1.53 mm) and *B. cereus* (20.33 ± 1.53 mm) and maintained activity against all test strains. Other isolates, such as ZN06 and TT03, exhibited selective but potent antimicrobial effects, particularly against *E. coli* (19.00 ± 1.73 mm and 18.33 ± 0.58 mm, respectively). In contrast, certain isolates, such as DMK01, DMK02, and ZN04, demonstrated limited activity, with inhibition observed only against a few test strains. Notably, ZN04 showed activity only against *S. aureus* (8.67 ± 0.58 mm) and *B. nealsonii* (11.33 ± 1.53 mm), while being inactive against other test strains. Overall, isolates HM05, K101, and TT02 emerged as the most promising candidates due to their broad-spectrum antimicrobial activity, indicating their potential as probiotic strains with antagonistic properties against pathogenic bacteria.

**TABLE 4:** Antagonistic activity of bacterial isolates against pathogenic organisms.

Bacterial isolates	Zone of inhibition (in mm)				
	*E. bugandensis* strain GCC_21R10	*B. cereus* strain GCC_21R1	*E. coli* ATCC 25922	*S. aureus* ATCC 23235	*B. nealsonii* strain GCC_21R8
DMK01	0	0	10.67 ± 1.53	9.33 ± 1.53	10.67 ± 1.53
DMK02	0	0	10.33 ± 1.15	9.00 ± 0.00	11.00 ± 0.00
DMK03	17.33 ± 1.15	13.67 ± 1.53	0	11.67 ± 0.58	0
HM01	14.00 ± 0.00	12.33 ± 0.58	14.67 ± 2.08	9.33 ± 1.53	0
HM03	0	0	0	9.00 ± 2.00	11.00 ± 0.00
HM05	20.33 ± 1.53	20.67 ± 0.58	13.33 ± 1.15	11.33 ± 1.53	13.67 ± 1.53
K101	21.67 ± 1.53	20.33 ± 1.53	14.00 ± 1.00	10.00 ± 0.00	14.00 ± 0.00
K102	0	0	11.00 ± 0.00	8.67 ± 0.58	10.33 ± 2.08
K104	18.33 ± 0.58	10.00 ± 2.00	18.33 ± 0.58	0	9.67 ± 0.58
K105	13.33 ± 2.08	11.00 ± 1.00	14.33 ± 1.53	9.00 ± 0.00	0
TT02	20.33 ± 1.53	20.33 ± 2.52	14.00 ± 1.00	11.33 ± 1.53	14.00 ± 1.00
TT03	17.67 ± 0.58	10.00 ± 0.00	18.33 ± 0.58	0	9.67 ± 0.58
TT04	18.67 ± 1.15	9.33 ± 1.15	18.67 ± 2.08	0	9.67 ± 0.58
ZN01	18.33 ± 0.58	9.67 ± 1.53	18.33 ± 1.53	0	10.67 ± 1.15
ZN04	0	0	0	8.67 ± 0.58	11.33 ± 1.53
ZN06	17.67 ± 1.53	11.00 ± 1.00	19.00 ± 1.73	0	10.00 ± 0.00

Values are expressed as mean *pm* standard deviation (SD) from three independent experiments.

### 3.5 Evaluation of technological properties

#### 3.5.1 Proteolytic activity

In the present study, all isolated strains demonstrated an absence of proteolytic activity, as evidenced by the lack of clear zones surrounding their colonies upon incubation on skimmed milk agar medium.

#### 3.5.2 Lipolytic activity

The absence of a halo clear zone surrounding the colonies of all isolated strains indicates the lack of lipolytic activity.

#### 3.5.3 Amylolytic activity

None of the isolated strains exhibited a clear zone surrounding their colonies upon flooding the agar plates with a 1% w/v iodine solution, indicating negative results for the amylolytic test.

## 4 Discussion

In the present study, the isolation and identification of LAB strains from rice-based fermented products in Southern Assam, India, revealed a diverse microbial community comprising Gram-positive bacilli and cocci. Molecular characterization corroborated these findings, highlighting the prevalence of *Pediococcus pentosaceus*, *Limosilactobacillus fermentum*, *Lactiplantibacillus plantarum*, and others in traditional fermented foods (Devi et al., 2020; Das et al., 2019). In addition to preservation benefits, traditional lactic acid fermentation of rice-based products often used to make alcoholic beverages, such as Haria, Judima, Hor alank, Zau, and Zu, has many other benefits. These findings highlight the importance of indigenous fermented foods as reservoirs of beneficial LAB strains with potential probiotic properties.

Our study assessed the probiotic potential of the isolated LAB strains was assessed using various criteria, including low pH, gastric juice, bile, pancreatin tolerance, cell surface hydrophobicity assay, and auto-aggregation assay. Our findings demonstrated statistically significant robust acid tolerance among several strains at pH 2 and pH 3, indicating their capacity to endure the harsh conditions of the gastrointestinal tract (GIT). The analysis reveals that DMK03 exhibited the most significant decrease in growth at pH 2, with a mean difference of 0.58 ± 0.05, indicating high sensitivity to extreme acidic conditions. At pH 3, ZN01 showed the highest decline, with a mean difference of 0.41 ± 0.01, suggesting reduced tolerance at moderately acidic conditions. On the other hand, ZN06 demonstrated the highest acid tolerance, with the least reduction in growth at both pH 2 (0.11 ± 0.07) and pH 3 (0.08 ± 0.06) compared to pH 7.2. Our results suggest that ZN06 has the highest potential for survival in the harsh acidic environment of the gastrointestinal tract. These results align with those reported by Islam et al. (2021) and Nath et al. (2020a) who observed similar acid tolerance in their studies. Furthermore, a study by Dharshini et al. (2021), found that all bacterial isolates exhibited resistance to various pH levels of 1.0, 2.0, and 3.0, which corroborates our findings.

Under gastric juice conditions, our isolates demonstrated varying survival rates after 3 h of incubation. Notably, TT03 (0.05 ± 0.18) and ZN06 (0.12 ± 0.26) exhibited the highest tolerance to gastric juice, showing the smallest reduction in mean values. These isolates demonstrate strong acid resistance, which is crucial for their potential probiotic applications in the gastrointestinal tract. On the other hand, isolates like K102 (1.37 ± 0.19) and HM03 (1.02 ± 0.12) showed the most significant decline in viability, suggesting lower resistance to acidic conditions. The variations in survival rates across different isolates highlight their differential tolerance, which could be useful for selecting robust probiotic strains capable of withstanding gastric acidity. These findings of the study are consistent with Mantzourani et al. (2019), who highlighted strain-dependent variations in survival rates. Additionally, Tokatlı et al. (2015) reported that specific strains could resist the acidic environment of gastric juice, further validating our observations. These findings underscore the importance of selecting probiotics that can withstand gastric juice for effective formulation (Urcan et al., 2024; Motta et al., 2022).

The present study also demonstrated that several isolates exhibited significant resistance to bile and pancreatin. Specifically, K101 showed the highest tolerance at 0.3% bile (0.07 ± 0.24), while TT02 demonstrated the highest tolerance at 0.5% bile (0.04 ± 0.15). ZN06 consistently performed best across both conditions, with mean differences of 0.29 ± 0.15 at 0.3% bile and 0.06 ± 0.16 at 0.5% bile. These results are consistent with findings by Abriouel et al. (2012), who demonstrated that the ability of LAB isolated from fermented olives could endure 0.3% bile salt. In the pancreatin tolerance test, the present study reported that HM05 exhibited the highest tolerance, with a mean difference of 0.43 ± 0.59, indicating its strong ability to survive digestive enzymatic conditions. In contrast, HM03 showed the lowest tolerance, with a mean difference of 2.08 ± 0.36, reflecting a substantial reduction in viability under pancreatin stress. Similar findings were reported by Dharshini et al. (2021), where LAB demonstrated diverse enzyme tolerance profiles.

Cell surface hydrophobicity and auto-aggregation were also assessed, which are critical for probiotic adherence and colonization in the GIT. The results of the present study revealed a wide range of hydrophobicity levels among the isolates, with several strains demonstrating strong affinity for hydrocarbons, suggesting enhanced gut mucosal adhesion. The variations in auto-aggregation indicate diverse adhesive properties, which may influence persistence in the host’s GIT (Bindu and Lakshmidevi, 2021). Additionally, variations in cellular auto-aggregation suggest diverse adhesive properties among the strains, potentially influencing their persistence and interaction with host epithelial cells (Malfa et al., 2023). Our study further revealed that several isolates could form biofilms to varying degrees. This characteristic, while beneficial for persistence in the GIT, poses a risk of contamination in food production environments. These observations align with findings by Gómez et al. (2016) and Emel et al. (2019). who emphasized the importance of balancing biofilm formation for probiotic efficacy without compromising food safety.

Antibiotic susceptibility profiling of the isolated strains demonstrated significant resistance to commonly used antibiotics. Notably, 16 isolates exhibited 100% resistance to co-trimoxazole and oxacillin, while 93.75, 87.5, and 81.25% of isolates were susceptible to gentamicin, amikacin, and norfloxacin, respectively. In a study by Temmerman et al. (2003), a total of 55 probiotic products from Europe were assessed to identify antibiotic resistance to vancomycin (65%), erythromycin (16%), and chloramphenicol (11%). In another study by Yerlikaya et al. (2020), all isolated *Lactobacillus bulgaricus* and *Streptococcus thermophilus* strains exhibited 100% resistance toward oxacillin (1 mcg) and nalidixic acid (30 mcg).

The safety assessment of potential probiotic strains is a critical prerequisite for their application in the food and health sectors. In the present study, all isolates tested negative for DNase activity, indicating the absence of extracellular deoxyribonucleases. DNase enzymes contribute to pathogenicity by degrading host DNA and facilitating evasion from neutrophil extracellular traps (Baz et al., 2024). Their absence supports the non-virulent nature of the isolates. According to Byakika et al. (2019) DNase-negative results are considered a key indicator of probiotic safety in LAB. Moreover, all isolates showed γ-hemolysis (no hemolysis) on blood agar, confirming the lack of hemolytic toxins such as cytolysins or pore-forming proteins. Hemolytic activity is a key virulence determinant, and its absence is a strong indicator of strain safety. This finding aligns with established safety benchmarks for probiotic lactic acid bacteria as outlined by Fanelli et al. (2023). The antagonistic activity of our LAB isolates against pathogenic organisms emphasizes their potential as biotherapeutic agents. Similar antimicrobial properties have been reported in LAB by other studies, where organic acids produced by LAB were shown to inhibit pathogen growth (Todorov et al., 2020; Barache et al., 2020).

In terms of technological properties, our isolates did not exhibit proteolytic, lipolytic, or amylolytic activities, indicating their suitability for use in food fermentation without compromising product quality or safety. These findings align with the desirable technological properties of probiotic LAB strains, emphasizing their potential for application in diverse food products (Pakroo et al., 2022). Thus, our findings provide valuable insights into the probiotic potential, safety profile, and technological properties of LAB strains isolated from traditional fermented foods in Southern Assam, India. These LAB strains exhibit significant tolerance to acid, bile, gastric juice, and pancreatin, along with strong adhesive properties, making them promising candidates for probiotic applications. Further research is warranted to elucidate their mechanisms of action and explore their applications in functional foods and nutraceuticals.

## 5 Conclusion

In the present study, a total of 16 lactic acid bacterial strains were isolated from fermented rice-based products, displaying significant promise as probiotic agents against gut pathogens. These isolates were identified, and the probiotic potential of the strains was evaluated through a series of *in vitro* tests, comprising gastric acid, acid, bile, pancreatin tolerance, exhibiting resilience and suitability for survival in the gastrointestinal tract, cell surface hydrophobicity, and auto-aggregation capabilities, indicating strong adhesion potential. Safety assessments established the isolates as safe for further application. This study highlights the importance of identifying and characterizing bacteria with probiotic properties, particularly in fermented rice-based products, as a potential source of beneficial probiotic bacteria for the ethnic tribes in Southern Assam. Our findings align with existing literature and demonstrate the significance of indigenous microbial resources in the development of functional foods and probiotic supplements. Further research is needed to uncover their mechanisms of action and explore their potential applications in functional foods and nutraceuticals.

## Data Availability

The datasets presented in this study can be found in online repositories. The names of the repository/repositories and accession number(s) can be found in the article/Supplementary material.
